# Chronic Stress Patterns Among Older Adults and Associations With Cognitive Functioning

**DOI:** 10.1093/geroni/igab046.2642

**Published:** 2021-12-17

**Authors:** Kun Wang, Zainab Suntai, Xiayu Chen

**Affiliations:** 1 University of Alabama, Tuscaloosa, Alabama, United States; 2 University of Illinois at Urbana-Champaign, New York, New York, United States

## Abstract

Chronic stress has been associated with several adverse cognitive outcomes, including impaired judgement, executive functioning, and memory. Chronic stress has also been linked to several neurological conditions, including Dementia and Alzheimer’s. While several biomedical measures of stress exist, stress is often subjective, and research has shown that the ability to cope with stress-known as stress reactivity-is more indicative of stress burden that the actual stressor itself. As such, this study aimed to identify the association between different patterns of stress and cognition among older adults. Data were derived from the 2016 Health and Retirement Study, a nationally representative study of older adults aged 50 and older living in the United States. Latent class analysis was used to identify different classes of stress and hierarchical linear regressions were conduced to identify the associations between identified stress classes and cognition. The latent class analysis resulted in four stress classes: high stress, financial stress, secondary stress, and low stress. The sequential logistic regression models revealed that while high stress and financial stress classes resulted in cognitive decline, the significance was mitigated after controlling for health and body functioning factors. This suggests that older adults are experiencing stressors mostly from health impairments and interventions should target improved health management and financial support for health conditions as an indirect way of reducing disparities in cognitive functioning resulting from chronic stress.

